# Yangjing Capsule Ameliorates Spermatogenesis in Male Mice Exposed to Cyclophosphamide

**DOI:** 10.1155/2015/980583

**Published:** 2015-12-21

**Authors:** Hongle Zhao, Baofang Jin, Xindong Zhang, Yugui Cui, Dalin Sun, Chao Gao, Yalong Gu, Bin Cai

**Affiliations:** ^1^Andrology Department of Integrative Medicine, Zhongda Hospital, School of Medicine, Southeast University, Nanjing 210009, China; ^2^Reproductive Medical Center, Department of Obstetrics and Gynecology, Nanjing Drum Tower Hospital, Nanjing University Medical School, Nanjing 210008, China; ^3^State Key Laboratory of Reproductive Medicine, Clinical Center of Reproductive Medicine, First Affiliated Hospital, Nanjing Medical University, Nanjing 210029, China

## Abstract

Yangjing capsule (YC), a traditional Chinese compound herbal preparation, has been proven as an effective drug to improve spermatogenesis in clinical practice. However, its pharmacological mechanisms were not fully clarified. This study was designed to investigate the protective effects of YC on spermatogenesis in the mouse model of spermatogenesis dysfunction induced by cyclophosphamide (CP). The administration of YC significantly increased the epididymal index, sperm count, and sperm motility of model mice. Histopathological changes demonstrated that CP caused obvious structural damage to testis, which were reversed by the administration of YC. Results from TUNEL assay showed that treatment with YC dramatically decreased the apoptosis of spermatogenic cell induced by CP. Moreover, YC treatment could inhibit the mRNA and protein expression of Bax to Bcl-2 and also raised expression of AR at both mRNA and protein levels. These data suggest that YC might ameliorate spermatogenesis in male mice exposed to CP through inhibiting the apoptosis of spermatogenic cell and enhancing the actions of testosterone in spermatogenesis.

## 1. Introduction

Infertility affects 15% of couples worldwide. It is estimated that roughly half of the infertility cases are due to male factors [1]. Male infertility could be caused by various reasons including failure in spermatogenesis, defects in sperm transportation or accessory gland function, genetic or environmental factors, and sexual disorders [2, 3]. Among these causes, spermatogenic defect is the primary one in male infertility [4].

Spermatogenesis is a sophisticated multistep process involving three major phases: mitotic, meiotic, and postmeiotic phases. Other cellular events such as apoptosis of spermatogenic cell, migration, and differentiation also play vital roles in the process of spermatogenesis [5]. The dynamic balance between cell proliferation and apoptosis determines the number of cells in the seminiferous tubules of the testis [6]. Excess apoptosis will result in depletion of sperm production. The whole process of spermatogenesis is also controlled by a series of hormones, in which testosterone plays a vital role in regulating spermatogenesis by binding to the androgen receptor (AR). The absence of testosterone or AR in the processes of spermatogenesis will weaken the actions of testosterone, resulting in dysfunction of spermatogenesis [7].

The Yangjing capsule (YC), a traditional Chinese compound herbal preparation, has been used for over ten years for the treatment of male reproductive diseases, in China, including male infertility and sexual dysfunction. Clinical practice showed that YC could improve the density of sperm, its motility, and DNA integrity in infertile men [8, 9]. These studies indicated that YC is an effective drug to improve spermatogenesis in infertile men, but the underlying mechanisms are not well understood. From in vitro experiments, it was found that YC could stimulate self-renewal of GC-1 spermatogonia cells and protect GC-1 spermatogonia cells from apoptosis [10]. It was also found that YC could promote the synthesis of testosterone in mouse Leydig tumor cells-1 by increasing the expression of steroidogenic acute regulatory protein (StAR), cytochrome P450, family 11, subfamily A, polypeptide 1 (CYP11A1), and 3-hydroxysteroid dehydrogenase/isomerase (3-HSD) mRNAs and proteins [11]. Therefore, it is assumed that YC treats the dysfunction of spermatogenesis by inhibiting the apoptosis of sperm cells and enhancing the synthesis and metabolism of testosterone. However, the effects of YC on testosterone synthesis, metabolism, and spermatogenic cell apoptosis are still unclear in vivo.

In recent years, mouse models of spermatogenesis dysfunction are used widely to investigate the mechanisms of spermatogenesis. Alkylating agents such as CP are the most common agents implicated in inducing the mouse model. Furthermore, it has been demonstrated that CP can inhibit testosterone synthesis and induce apoptosis of spermatogenic cells [12, 13]. Therefore, CP was selected to obstruct the spermatogenesis of mice in this project. This study is aimed at investigating the protective effects of YC on the spermatogenesis, testosterone synthesis, and apoptosis of spermatogenic cells in a mouse model of spermatogenesis dysfunction induced by CP.

## 2. Materials and Methods

### 2.1. Drugs and Chemicals

CP was purchased from Pude Medicine Co. Ltd. (Shanxi, China). Iodine [^125^I] Testosterone Radioimmunoassay Kit was supplied by Beijing North Institute of Biological Technology (Beijing, China). The primers were synthesized by Invitrogen Life Tech (Carlsbad, CA). The mouse monoclonal anti-glyceraldehyde 3-phosphate dehydrogenase (anti-GAPDH) and horseradish peroxidase-conjugated secondary antibodies were purchased from Bioworld (St. Louis Park, MN). The rabbit monoclonal anti-tubulin and rabbit polyclonal anti-Bax antibodies were procured from Abcam (Cambridge, MA). The rabbit polyclonal anti-AR and rabbit polyclonal anti-B-cell lymphoma 2 (anti-Bcl-2) antibodies, goat anti-rabbit immunoglobulin G (*γ*-chain specific) (peroxidase conjugate), and In Situ Cell Apoptosis Detection Kit I (POD) were procured from Boster Biological Engineering Co. Ltd. (Wuhan, China).

### 2.2. Preparation of the YC

The YC was provided by Nanjing General Hospital of Nanjing Military Region (Nanjing, China). YC is composed of 11 types of traditional Chinese drugs: 13.3% Herba Epimedii Brevicornus, 6.7% Rhizoma Polygonati Sibirici, 6.7% Radix Rehmanniae Preparata, 10% Radix Astragali Mongolici, 6.7% Placenta Hominis, 6.7% Semen Astragali Complanati, 10% Radix Angelicae Sinensis, 6.7% Hirudo, 6.7% Semen Litchi, 13.3% Semen Vaccariae Segetalis, and 13.3% Concha Ostreae (calcined). In this study, contents of the dried capsule were diluted into different concentrations using distilled deionized water, and the suspensions were stored at 4°C.

### 2.3. Animals and Treatments

Mature male (8-week) Balb/c mice (22–25 g) were procured from Yangzhou University Comparative Medical Center (Yangzhou, China). Mice were housed under standard laboratory conditions and were provided with free access to standard food and water ad libitum. This study was approved by the Animal and Human Ethics Board of Southeast University. After adapting to the environment for a week, mice were randomly divided into four groups: control (*n* = 9), CP (*n* = 9), CP plus YC (630 mg/kg) (*n* = 9), and CP plus YC (1260 mg/kg) (*n* = 9). For the first 7 days, the mice of CP, CP plus YC (630 mg/kg), and CP plus YC (1260 mg/kg) groups were injected intraperitoneally (i.p.) with 50 mg/kg of CP once a day. This method has been previously shown to induce dysfunction of spermatogenesis [14]. For the subsequent 30 days, the mice in the YC treatment groups were administered with YC suspension once a day by oral gavage. The animals were weighed weekly to adjust the gavage volume, and their general health was monitored daily.

### 2.4. Preparation of Serum and Tissue

The animals were weighed and anesthetized with pentobarbital sodium (50 mg/kg, i.p.) at the end of the treatment. Blood samples were collected by extirpation of eyeball and allowed to clot, and the serum was separated at 3000 rpm for 15 min and stored at −80°C to analyze the testosterone level.

The testes and epididymis were rapidly excised and weighed. The relative testicular or epididymal weight (the testis index or the epididymal index) was calculated by dividing the weight of testis or epididymis by body weight. One testis and one epididymis from a mouse were fixed in Bouin's solution for pathological examination and terminal deoxynucleotidyl transferase dUTP nick end labeling (TUNEL) analysis. The other testis was freshly washed in ice-cold saline and immediately stored in liquid nitrogen. The fresh testis frozen in liquid nitrogen was then divided into two parts for the isolation of RNA and extraction of protein.

### 2.5. Epididymal Sperm Analysis

The epididymal sperm concentrations and motility were evaluated as previously described with some modifications [15]. An entire epididymis from a mouse was minced in 1 mL saline, which was preheated at 37°C. The epididymis was incubated for another 15 min at 37°C to allow the sperm to swim out of the epididymal tubules. The total number of sperm and the number of motile sperms were determined using a hemocytometer (Anxin, Shanghai, China); the sperm motility was converted into a percentage.

### 2.6. Histological Examination

For histological studies, tissues were initially fixed in Bouin's fluid for 16 h and they were then washed three times with 70% ethanol followed by dehydration in graded ethanol. They were finally embedded in paraffin. Five-micrometer-thick sections were stained using hematoxylin-eosin and then examined under a light microscope at 200x or 400x magnification. A total of 200 seminiferous tubules from each testis were examined. Damaged tubules which showed severe hypocellularity (reduction in number of spermatogenic cells) and thinned seminiferous epithelium were recorded.

### 2.7. Assessment of Testosterone

Serum testosterone was examined using Iodine [^125^I] Testosterone Radioimmunoassay Kit according to the protocol described by the manufacturer. The amount of testosterone was determined from a calibration curve.

### 2.8. In Situ Detection of Apoptosis

Five-micrometer-thick sections of the testis were used for TUNEL staining to examine cell apoptosis. The TUNEL method was performed according to the manufacturer's instructions. The numbers of positive and total cells were recorded, and the apoptosis index [(number of positive cells/total cells) × 100] of the tissue was calculated as described by Yuan et al. [14].

### 2.9. Isolation of RNA and Real-Time Quantitative PCR

Bax, Bcl-2, and AR mRNA expression levels in the testes were determined using real-time quantitative PCR. The total RNA of the testes samples was extracted using Trizol reagent (TaKaRa Biotechnology, Dalian, China). Sample cDNAs were used as templates for amplification to quantify the mRNA levels of target genes using SYBR Green PCR Master Mix reagent kits (TaKaRa Biotechnology). GAPDH was selected as the control. The primer sequences were as follows: GAPDH: forward, 5′-TGG CCT TCC GTG TTC CTA C-3′; reverse, 5′-GAG TTG CTG TTG AAG TCG CA-3′; AR, forward, 5′-TCC AAG ACC TAT CGA GGA GCG-3′; reverse, 5′-GTG GGC TTG AGG AGA ACC AT-3′; Bax, forward, 5′-AGA CAG GGG CCT TTT TGC TAC-3′; reverse, 5′-AAT TCG CCG GAG ACA CTC G-3′; Bcl-2, forward, 5′-GCT ACC GTC GTG ACT TCG C-3′; reverse, 5′-CCC CAC CGA ACT CAA AGA AGG-3′. Relative quantification of expression of target genes was estimated using the 2^−ΔΔCt^ method.

### 2.10. Western Blot Analysis

Total proteins extracted from testis were prepared following standard procedures and quantified using the bicinchoninic acid protein assay (Beyotime, Shanghai, China). The proteins were separated by 12% sodium dodecyl sulfate-polyacrylamide gel electrophoresis and subsequently transferred onto nitrocellulose membranes. The membranes were blocked with 5% skim milk in Tris-buffer saline containing 0.1% Tween 20 at 37°C for 1 h and then incubated at 4°C overnight with the primary antibodies of Bax (1 : 200 dilution), Bcl-2 (1 : 300 dilution), and AR (1 : 300 dilution). Tubulin or GAPDH was used as an internal control. Densitometric analysis of the scanned immunoblotting images was performed using a Quantity One image system.

### 2.11. Statistical Analysis

All quantitative data derived from this study were analyzed using the SPSS 20.0 statistical package. The results were expressed as the mean ± standard deviation. The one-way analysis of variance was used to analyze the difference between groups, and post hoc least significant difference test was used for intergroup comparisons. *P* < 0.05 was considered as statistical significance.

## 3. Results

### 3.1. Effects of YC on the Relative Weights of Reproductive Organ and the Sperm Parameters

The relative weights of reproductive organ and the sperm parameters were used to monitor the damage to the male reproductive system. As shown in Table 1, in model mice treated with CP, there were significant decreases in body weight, the testis index, and the epididymal index when compared with the control group (*P* < 0.01, resp.). CP treatment also decreased the sperm count and motility by 47% and 34%, respectively, as compared to the control group (*P* < 0.01). After treatment with YC, the body weight and the testis index in the YC (1260 mg/kg) group significantly increased by 8.1% and 20.7% as compared to the CP group. YC (630 and 1260 mg/kg) treatments also significantly increased the epididymal index by 30.9% and 24.5% (*P* < 0.01) when compared with the CP-treated group. YC (630 and 1260 mg/kg) treatment also notably increased the sperm count and motility when compared with the CP group (Table 1).

### 3.2. Treatment with YC Reduced the CP-Induced Testicular and Epididymal Injury in Model Mice

**Figure 1:**
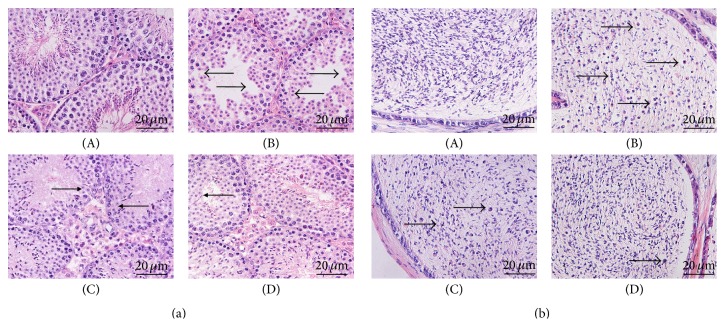
The effects of treatment with YC on the testicular ((a), ×400) and epididymal ((b), ×400) damage in the spermatogenesis dysfunction mice induced by CP (lesion site labeled with arrow heads). (A) control; (B) CP (50 mg/kg); (C) CP + YC (630 mg/kg); (D) CP + YC (1260 mg/kg).

The histopathology of the testis and epididymis was shown in Figure 1. Normal histological features of testis were visible in the control group: the normal thickness of the seminiferous epithelial layer, a number of spermatogenic cells, and interstitial cells (Figure 1(a)(A)). The epididymis tissues had normal density of sperm and few premature sperm cells (Figure 1(b)(A)). In contrast, as shown in Figure 1(a)(B), the CP-treated mice displayed the atrophied seminiferous tubules, the thinned seminiferous epithelium that was arrayed loosely, the reduced spermatogenic cells that were shed particularly in the postmeiotic stages, and the reduced interstitial cells. The administration of CP also lowered the density of sperm and increased the number of premature sperm cells which came off from the seminiferous tubules in cauda epididymis. These results were in agreement with a previous study [16] (Figure 1(b)(B)). The abovementioned histological damage instances were restored at least in some degree by treatment with YC.

In the YC (630 mg/kg) group, in spite of thinned seminiferous epithelium, many mature spermatids appeared in the seminiferous tubules (Figure 1(a)(C)). In the YC (1260 mg/kg) group, the layer of seminiferous epithelium showed a significant increase. The sperm cells at different stages were distributed regularly, and many mature spermatids appeared in the seminiferous tubules (Figure 1(a)(D)). In addition, the number of interstitial cells in the YC-treated groups presented a significant increase (Figures 1(a)(C) and 1(a)(D)). YC (630 and 1260 mg/kg) treatments also improved the density of sperms and decreased the number of premature sperm cells in cauda epididymis (Figures 1(b)(C) and 1(b)(D)).

The mean percentage of damaged seminiferous tubules in the CP-treated mice was significantly higher, 75.1 ± 5.3%, which was 2.9 ± 2.3% in the control group (Table 1). After daily administration of YC, the rates of damaged tubules dropped significantly, 20.5 ± 7.6% in the YC (630 mg/kg) and 13.3 ± 6.1% in the YC (1260 mg/kg) group.

### 3.3. Effect of YC on Serum Testosterone Level

The administration of CP caused a significant decrease in the serum testosterone level, from 1.81 ± 0.86 mol/L in the control group to 0.56 ± 0.22 mol/L in the CP-treated group (*P* < 0.05). In the YC (630 and 1260 mg/kg) groups, the values were 0.61 ± 0.26 and 0.76 ± 0.35 (mol/L), respectively. Although the serum testosterone level increased in the YC-treated groups, the difference did not reach statistical significance when compared with the CP group (Table 1).

### 3.4. Treatment with YC Inhibited CP-Induced Apoptosis of Spermatogenic Cell

**Figure 2:**
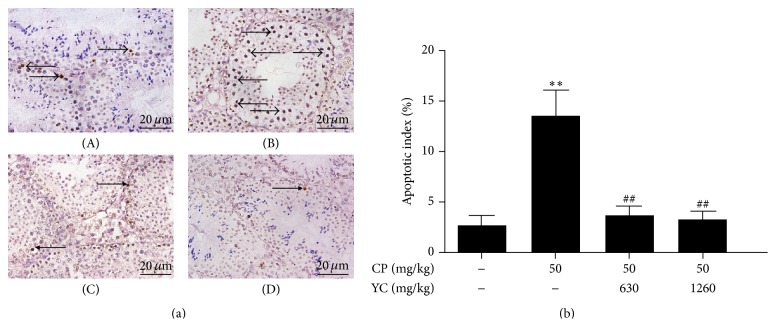
The effects of treatment with YC on apoptosis of spermatogenic cell in the spermatogenesis dysfunction mice induced by CP. (a) The representative apoptosis of spermatogenic cell in the testes using TUNEL assay (×400). (b) The spermatogenic cell apoptotic index. (A) Control; (B) CP (50 mg/kg); (C) CP + YC (630 mg/kg); (D) CP + YC (1260 mg/kg) (*n* = 5); ^*∗∗*^ *P* < 0.01 compared to the control group; ^##^ *P* < 0.01 compared to the CP-treated group. CP: cyclophosphamide; TUNEL: terminal deoxynucleotidyl transferase dUTP nick end labeling; YC: Yangjing capsule.

TUNEL assay was performed to analyze the apoptosis of spermatogenic cell. After administration of CP, apoptotic cells were observed more frequently in the testis of the CP group mice (Figure 2(a)(B)) than those in the control group (Figure 2(a)(A)). The apoptotic cells in the CP group were mainly spermatocytes. After treatment with YC, fewer apoptotic cells were detected in the testis sections (Figures 2(a)(C) and 2(a)(D)). The administration of CP also resulted in a significant increase in the apoptotic index of spermatogenic cell (13.4 ± 2.6%), compared to 2.5 ± 1.0% in the control group (Figure 2(b)). When treatment was carried out with YC (630 and 1260 mg/kg), there was a remarkable decrease in the apoptotic index by 72.6% and 75.4%, respectively, compared with the CP group (*P* < 0.01).

### 3.5. Effects of YC on Bax, Bcl-2 mRNA, and Protein Expression

**Figure 3:**
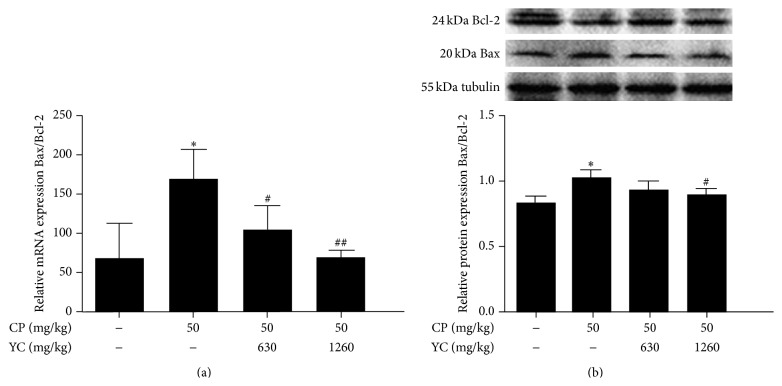
The effects of YC on the ratio of Bax to Bcl-2 mRNA expression (a) and protein expression (b) in the testes of spermatogenesis dysfunction mice induced by CP. Data given are the mean ± SD (*n* = 4). ^*∗*^ *P* < 0.05 compared to the control group; ^#^ *P* < 0.05, ^##^ *P* < 0.01 compared to the CP-treated group. CP: cyclophosphamide; SD: standard deviation; YC: Yangjing capsule.

To further study the potential mechanism of antiapoptosis of YC, the expressions of Bax and Bcl-2, at mRNA and protein levels, in the testes of mice were examined using the quantitative real-time PCR and Western blotting. As shown in Figure 3, the testes from the CP group mice showed a significant increase in the ratio of Bax to Bcl-2 mRNA and protein expression levels as compared to the control group (*P* < 0.05, resp.). Treatment with YC significantly decreased the ratio of Bax mRNA to Bcl-2 mRNA in a dose-dependent manner (Figure 3(a)), while YC (1260 mg/kg) dramatically reduced the ratio of Bax to Bcl-2 protein when compared with the CP-treated group (Figure 3(b)) (*P* < 0.05).

### 3.6. Effects of YC on AR mRNA and Protein Expression

**Figure 4:**
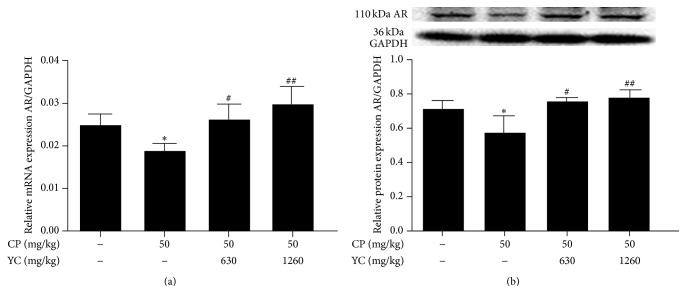
The effects of YC on AR mRNA (a) and protein expressions (b) in the testes of spermatogenesis dysfunction mice induced by CP. Data are expressed as mean ± SD (*n* = 3). ^*∗*^ *P* < 0.05 compared to the control group; ^#^ *P* < 0.05, ^##^ *P* < 0.01 compared to the CP-treated group. AR: androgen receptor; CP: cyclophosphamide; GAPDH: glyceraldehyde 3-phosphate dehydrogenase; SD: standard deviation; YC: Yangjing capsule.

Testosterone is the major androgen in the testis that regulates spermatogenesis. Effects of androgen are mediated by the AR. The lowered testosterone or dysfunctional AR will result in the impaired spermatogenesis. In this study, the expressions of AR mRNA and protein in the testes of different groups were compared. As shown in Figure 4, administration of CP significantly decreased the expressions of AR mRNA and protein (*P* < 0.05, resp.) as compared with the control group. YC (630 and 1260 mg/kg) treatment significantly improved the AR mRNA and protein expression levels when compared with the CP group (Figures 4(a) and 4(b)).

## 4. Discussion

Male infertility causes significant duress to couples. Defects in spermatogenesis are the most common reasons for male infertility. At present, hormonal treatment or empiric medical treatment (e.g., aromatase inhibitor or selective estrogen receptor modulator) is used to treat infertile men with spermatogenic defects, but clinical results are limited especially for patients with idiopathic failure of spermatogenesis [17]. Traditional Chinese formulations, intended for kidney support, have shown remarkable advantages in the treatment of male infertility [18]. YC, a Chinese compound herbal preparation, has been proven as an effective drug to improve spermatogenesis in the clinical practice for over ten years, but the underlying mechanisms are not well understood. Therefore, the present study was performed to study the effects of YC on the spermatogenesis and the underlying mechanisms in a mouse model of spermatogenesis dysfunction.

CP, as an alkylating agent, is the most common agent implicated in causing dysfunction of spermatogenesis. In this study, mature male Balb/c mice were injected with CP (50 mg/kg) i.p. for 7 days to induce the dysfunction of spermatogenesis. For the next 30 days, the mice received treatment with YC. Animals were killed at 30 days after the last injection. We found that administration of CP significantly decreased the sperm count and motility as well as the relative testicular weights and body weights. The administration of CP also caused histologic lesions of the testis and epididymis. Testicular tissues from the CP-treated mice showed atrophied seminiferous tubules, thinned seminiferous epithelium, and reduced spermatogenic cells and interstitial cells. We also found that the cauda epididymis from the CP-treated mice illustrated a lower density of sperm and an increase in the number of premature sperm cells.

Interestingly, we found that treatment with YC significantly restored these CP damage instances. YC (630 and 1260 mg/kg) treatment not only notably increased the sperm count and motility but also increased seminiferous epithelial layers and the number of mature spermatids and interstitial cells in testis. YC treatment also increased the density of sperm and decreased the deciduous premature sperm cells in the ductus epididymis when compared with the CP group. These results indicated that YC could ameliorate spermatogenesis in male mice exposed to CP.

Mammalian spermatogenesis depends on the balance among proliferation, differentiation, and apoptosis of germ cells. Apoptosis of spermatogenic cell occurs during various stages of mammalian spermatogenesis to remove abnormal spermatogenic cells [6], which maintains the ratio of germ cell to Sertoli cell within a normal range so as to keep the continual spermatogenesis [19]. However, excess apoptosis will be followed by severe impairment in the production of sperm. Among those modulators of apoptosis, the Bcl-2 subfamily (Bcl-2, Bcl-x long, Bcl-w, Mcl-1, A1/Bfl1, and Nr13) promotes the survival of cell and the Bax subfamily (Bax, Bcl-x short, Bak, and Bok) enhances the apoptosis of cell [19]. The ratio of Bax to Bcl-2 has been implicated as a critical determinant of cell fate [20]. It has been reported that acute or chronic exposure to CP results in elevated apoptotic rates of germ cell [21, 22]. Studies have revealed that the elevated apoptotic rate of germ cell induced by CP was related to the elevated ratio of Bax to Bcl-2 [14, 23]. In the present study, administration of CP significantly increased TUNEL-positive cells in the testis sections. Administration of CP also increased the ratio of Bax to Bcl-2 mRNA and protein expression levels in the testes of mice. After remedial treatment with YC, the increased apoptotic sperm cells and apoptotic index by CP treatment were dramatically reduced, accompanied with the reduction in the ratio of Bax to Bcl-2 mRNA and protein expression levels when compared with the CP group. These results indicated that YC inhibited the CP-induced apoptosis of spermatogenic cells by normalizing the elevated ratio of Bax to Bcl-2.

Testosterone is a major regulator in spermatogenesis, which works by binding to AR. In the absence of testosterone or the dysfunctional AR, spermatogenesis is halted during meiosis so that few germ cells develop to the haploid spermatid stage and the elongated spermatid can not form [24–26]. Furthermore, in testosterone-suppressed rats or AR hypomorphic mice, the attachment of Sertoli cells to round spermatids or elongated spermatids could not be maintained and germ cells are released prematurely [27, 28]. In addition, mature spermatids are retained and phagocytized by Sertoli cells in the absence of testosterone signaling [24]. The absence of testosterone or AR in the processes of spermatogenesis will weaken the actions of testosterone, resulting in the dysfunction of spermatogenesis.

Previous studies have shown that administration of CP could inhibit the synthesis of testosterone [12, 29, 30]. In this study, administration of CP caused a significant decrease in the serum testosterone level, which was consistent with previous studies. In addition, CP also markedly lowered the expressions of AR mRNA and protein in the testes when compared with the control group. The decreased testosterone level and AR expression level in the CP group probably contributed to the morphological injuries of testis and epididymis. After treatment with YC, serum testosterone level displayed a rising trend in the model mice, although this difference did not reach statistical significance as compared with the CP group. However, treatment with YC substantially upregulated the expression of AR when compared with the CP group. These indicated that YC may enhance the actions of testosterone to improve spermatogenesis.

As we know, traditional Chinese medicine, especially those compound recipes, plays a role through multiway and multitarget with its multicomponents. YC is composed of 11 traditional Chinese drugs. Of necessity, YC contains many active ingredients. It has been demonstrated that total flavonoids of Herba Epimedii Brevicornus and polysaccharides could not only enhance testosterone actions [11, 31], but also inhibit cell apoptosis [11, 14]. In addition, it has been reported that flavonoids and polysaccharides exerted clear antioxidative activities [11, 14]. Oxidative stress is an important cause of male infertility, which can increase the apoptosis of germ cells and decrease the synthesis of testosterone [32]. Therefore, the protective effects of YC on spermatogenesis may be related to its antioxidant effect. Based on the above analysis, we believe that the active ingredients in YC which ameliorate spermatogenesis could be total flavonoids of Herba Epimedii Brevicornus and polysaccharides. Additional studies for the pharmacological mechanisms of YC are necessary because this innovative traditional Chinese medicine has complex active ingredients.

## 5. Conclusions

In conclusion, the present study suggests that treatment with YC can ameliorate the impairment of spermatogenesis induced by CP in male mice. The effects of YC on improving spermatogenesis are likely due to the inhibition of apoptosis of spermatogenic cell and enhancement in the actions of testosterone in spermatogenesis. Therefore, YC might be considered as an alternative therapeutic remedy for male infertility.

## Figures and Tables

**Table 1:** Effects of YC on characteristics of sperm, seminiferous tubules, serum testosterone, body weight, and the relative testicular and epididymal weights.

Parameters	Control	CP	CP + YC (630 mg/kg)	CP + YC (1260 mg/kg)
Sperm count (×10^6^ mL^−1^)	82.56 ± 7.49	43.94 ± 4.70^*∗∗*^	51.31 ± 2.67^#^	65.11 ± 8.18^##^
Sperm motility (%)	69.00 ± 6.22	45.76 ± 7.28^*∗∗*^	62.22 ± 4.68^##^	65.25 ± 7.06^##^
Proportion of damaged tubules (%)	2.9 ± 2.3	75.1 ± 5.3^*∗∗*^	20.5 ± 7.6^##^	13.3 ± 6.1^##^
Body weight (g)	29.11 ± 1.54	26.0 ± 1.41^*∗∗*^	26.50 ± 1.41	28.11 ± 1.76^##^
Relative testicular weight (mg/g bodyweight)	4.02 ± 0.28	2.75 ± 0.54^*∗∗*^	3.13 ± 0.29	3.32 ± 0.39^##^
Relative epididymal weight (mg/g bodyweight)	1.47 ± 0.20	1.10 ± 0.11^*∗∗*^	1.44 ± 0.13^##^	1.37 ± 0.12^##^
Testosterone (mol/L)	1.81 ± 0.86	0.56 ± 0.22^*∗*^	0.61 ± 0.26	0.76 ± 0.35

Data are expressed as the mean ± SD (*n* = 9). ^*∗*^ *P* < 0.05, ^*∗∗*^ *P* < 0.01 compared to the control group; ^#^ *P* < 0.05, ^##^ *P* < 0.01 compared to the CP-treated group.

CP: cyclophosphamide; SD: standard deviation; YC: Yangjing capsule.

## Abbreviations

YC: Yangjing capsule
CP: Cyclophosphamide
TUNEL: Terminal deoxynucleotidyl transferase dUTP nick end labeling
AR: Androgen receptor
StAR: Steroidogenic acute regulatory protein
CYP11A1: Cytochrome P450, family 11, subfamily A, polypeptide 1
3*β*-HSD: 3*β*-Hydroxysteroid dehydrogenase/isomerase
Bax: Bcl-2 Associated X Protein.
